# Immune, inflammatory, and metal biomarker profiles in chronic respiratory diseases receiving oligo-fucoidan under ambient PM₂.₅ exposure

**DOI:** 10.1038/s41598-026-42873-6

**Published:** 2026-03-13

**Authors:** Juei-Nan Cho, Chia-Ju Shih, Tu-Chen Liu, Ya-Ling Chiou

**Affiliations:** 1 Department of Nutrition (Master Program), Hungkuang University, 34 Chung-Chie Rd, Sha Lu, Taichung, Taiwan, Republic of China; 2 Department of Chest Medicine, Cheng-Ching General Hospital, Taichung, Republic of China; 3 Department of Medical Research, Kuang Tien General Hospital, Taichung, Republic of China

**Keywords:** PM_2.5_, chronic respiratory diseases, oligo-fucoidan, immune responses, inflammation, Diseases, Environmental sciences, Immunology, Medical research

## Abstract

Industrial zones are prevalent in central Taiwan, with major sources of ambient air pollution including large thermal power plants and steel manufacturing facilities. Fine particulate matter (PM₂.₅) is a critical component of air pollution and has been implicated in the development and progression of chronic respiratory diseases (CRDs), primarily through pathways involving immune dysregulation and persistent low-grade inflammation. Oligo-fucoidan (OF), a low–molecular-weight derivative of fucoidan, has been reported to exert immunomodulatory and anti-inflammatory effects in experimental and preclinical studies. However, clinical evidence regarding its potential role in air pollution–associated respiratory conditions remains limited. This exploratory, non-randomized, open-label study aimed to descriptively evaluate changes in immune and inflammatory parameters among patients with air pollution–associated chronic pulmonary diseases residing in central Taiwan. A total of 46 participants received OF supplementation (2.2 g daily) for 12 weeks in addition to standard care. Blood samples were collected at baseline and at weeks 4, 8, and 12 to assess biochemical indices, lymphocyte subsets, inflammatory cytokines, and serum heavy metal concentrations. Ambient PM₂.₅ data during the study period were obtained from nearby governmental air quality monitoring stations. Ambient PM₂.₅ concentrations during the study period were within a relatively low range (11.1 ± 4.0 to 14.7 ± 10.3 µg/m³). Over the 12-week supplementation period, descriptive variations were observed in several immune and inflammatory markers, including white blood cell count, C-reactive protein, ferritin levels, lymphocyte subset distributions, and selected cytokines. Serum mercury concentrations demonstrated a positive association with ambient PM₂.₅ levels. Among the measured cytokines, IL-8 values at later time points were lower than at baseline; these changes are presented descriptively and should be interpreted cautiously given the exploratory, single-arm nature of the study. This exploratory study provides a descriptive characterization of immune, inflammatory, and heavy metal–related parameters in patients with air pollution–associated chronic pulmonary diseases receiving oligo-fucoidan as an adjunct to standard therapy. While causal relationships and statistically confirmed longitudinal effects cannot be established, these preliminary observations may inform the design of future controlled trials and mechanistic investigations in the context of environmental respiratory health.

## Introduction

Chronic respiratory diseases (CRDs) represent a major global health burden. According to the 2019 Global Burden of Disease (GBD) study, approximately 455 million individuals worldwide (95% uncertainty interval [UI]: 417–499 million) were affected by CRDs, reflecting an increase of nearly 40% since 1990^1^. CRDs comprise a heterogeneous group of conditions, including chronic obstructive pulmonary disease (COPD), asthma, occupational lung diseases, and other chronic pulmonary disorders^2^. Although these conditions are generally incurable, appropriate management strategies can alleviate symptoms and improve quality of life. Established risk factors for CRDs include tobacco and secondhand smoke exposure, indoor and outdoor air pollution, occupational hazards, early-life respiratory infections, and genetic susceptibility^3^.

Airborne particulate matter (PM), particularly fine particles with aerodynamic diameters ≤ 2.5 μm (PM₂.₅), is a major environmental contributor to the development and progression of CRDs^4–6^. PM can be categorized as primary particles emitted directly from natural or anthropogenic sources, or secondary particles formed through atmospheric chemical reactions^7^. Natural sources include windblown dust, sea spray, forest fires, and volcanic activity, whereas anthropogenic sources include industrial emissions, coal-fired power plants, waste incineration, and vehicular exhaust. Secondary PM₂.₅ is largely generated through the transformation of sulfur oxides (SOₓ), nitrogen oxides (NOₓ), and volatile organic compounds into sulfates, nitrates, ammonium salts, and carbonaceous particles^8,9^.

Due to its small size, PM₂.₅ can penetrate deeply into the respiratory tract, reach the alveoli, and translocate into the systemic circulation^10,11^. PM₂.₅ contains toxic constituents, including heavy metals and reactive chemical species, which induce oxidative stress, endoplasmic reticulum stress, and inflammatory responses in pulmonary tissues^12,13^. These processes have been implicated in the exacerbation of respiratory and cardiovascular diseases, metabolic dysfunction, and carcinogenesis. Epidemiological and experimental studies have further linked PM₂.₅ exposure to impaired alveolar macrophage function, increased asthma severity, reduced lung function, and elevated lung cancer risk^14–16^. Mechanistically, PM₂.₅-induced respiratory injury is mediated in part by activation of oxidative stress–responsive and inflammatory signalling pathways, including nuclear factor kappa B (NF-κB) and mitogen-activated protein kinases (MAPKs), resulting in increased production of pro-inflammatory cytokines such as tumor necrosis factor alpha (TNF-α) and interleukin-8 (IL-8). Chronic exposure may also disrupt immune homeostasis by altering lymphocyte populations, thereby contributing to sustained airway inflammation and disease progression^17–19^.

Fucoidan is a sulfated polysaccharide derived from brown seaweed and has been reported to possess a range of bioactive properties, including anti-inflammatory, antioxidant, antiviral, antimicrobial, anticoagulant, and antitumor effects^20–23^. Experimental studies, including animal models, have demonstrated that fucoidan can attenuate PM₂.₅-induced allergic and inflammatory responses in the respiratory system^24^, suggesting a potential role in modulating air pollution–related pulmonary inflammation. Mechanistically, fucoidan has been shown to inhibit NF-κB activation, suppress MAPK phosphorylation, and reduce the production of pro-inflammatory cytokines in cellular and animal models of airway inflammation. In addition, fucoidan has been widely consumed as a functional food ingredient and dietary supplement, with human studies in metabolic, inflammatory, and oncological contexts reporting favorable tolerability and safety profiles. Although clinical evidence specifically targeting chronic respiratory diseases remains limited, these observations support the feasibility of further clinical evaluation^25–27^.

Oligo Fucoidan (OF) is a sulfated fucose-rich polysaccharide that is isolated from the brown seaweed Laminaria japonica (Saccharina japonica), using enzymatic hydrolysis process to extract high purity small-molecular-weight fucoidan, which enhances intestinal absorption and may improve biological activity compared with native high–molecular-weight fucoidan^28–30^. Owing to its improved bioavailability and standardized composition, OF was selected as the investigational formulation in the present study. OF was administered orally at a daily dose of 2.2 g, a regimen previously reported to be safe in human supplementation studies^31,32^.

In this exploratory study, we evaluated changes in immune, inflammatory, and heavy metal–related parameters following 12 weeks of OF supplementation in 46 patients with chronic pulmonary diseases residing in areas affected by ambient air pollution. Peripheral blood samples were collected at baseline and at weeks 4, 8, and 12 to assess biochemical indices, lymphocyte subsets, inflammatory cytokines, and serum heavy metal concentrations. This study aimed to provide preliminary clinical observations to inform future controlled trials and mechanistic investigations into adjunctive interventions for air pollution–associated chronic respiratory diseases.

## Materials and methods

### Study subjects

**Fig. 1:**
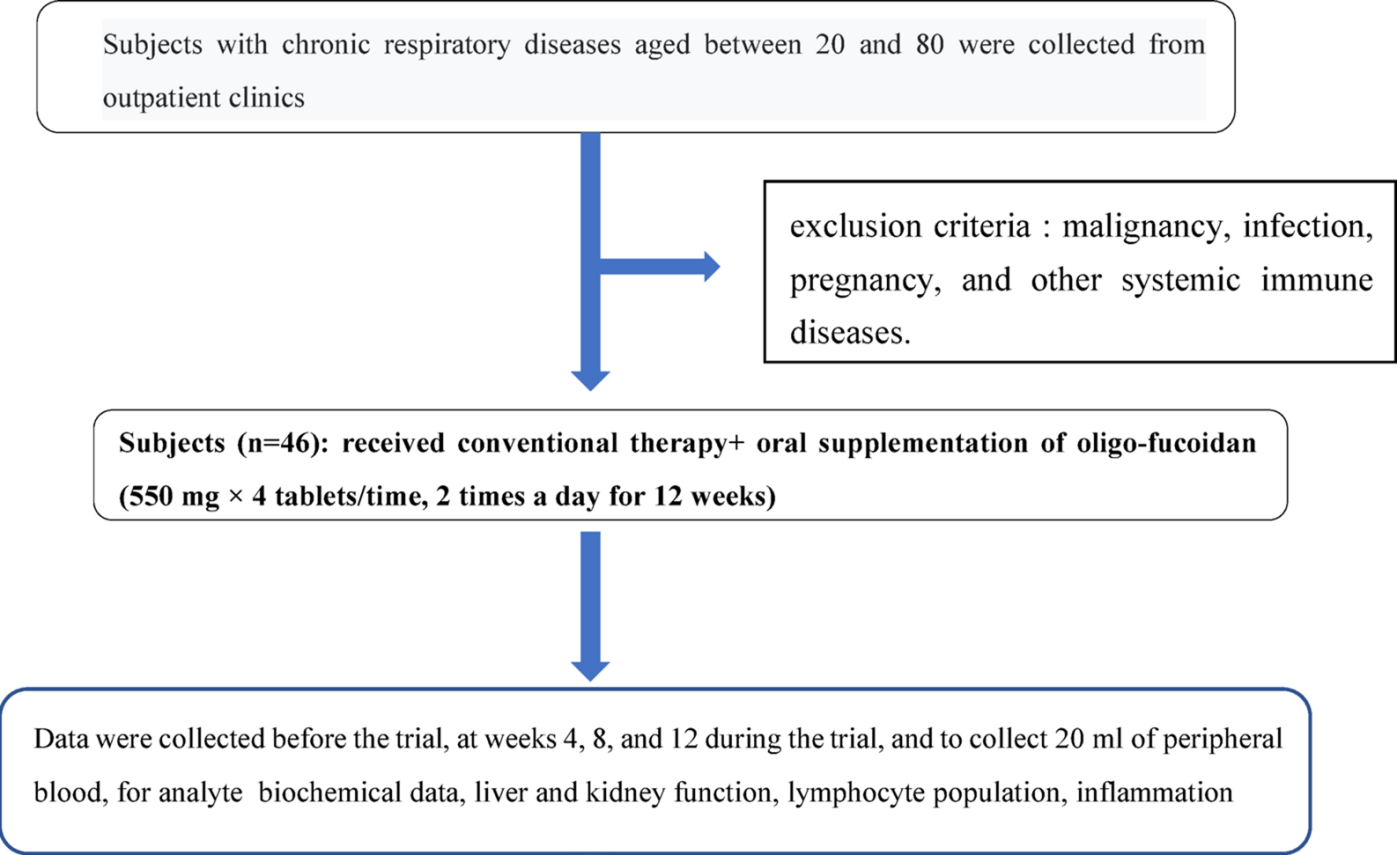
Flowchart for collecting subjects.

This study was conducted as a non-randomized, open-label, single-arm exploratory clinical investigation. Randomization and blinding were not implemented due to the exploratory nature of the study and the absence of a parallel control group. No major protocol deviations, including changes in intervention dosage or study procedures, were identified during the study period. A total of 46 participants were recruited from the outpatient clinics of the Department of Pulmonology at Cheng Ching Hospital and the Sha Lu Chao An Clinic. Inclusion criteria were as follows:^1^ male or female participants aged 20–80 years at the time of screening, and^2^ a documented history of chronic pulmonary disease, including chronic bronchitis, emphysema, asthma, or chronic obstructive pulmonary disease (COPD). Exclusion criteria included malignancy, active infection, pregnancy, use of immunosuppressive agents other than corticosteroids or bronchodilators, and systemic immunological disorders (Fig. 1). Oligo-Fucoidan (OF)n is a sulfated fucose-rich polysaccharide that is isolated from the brown seaweed Laminaria japonica (Saccharina japonica), using Hi-Q Marine Biotech’s advanced (enzymatic) hydrolysis process to extract high purity small-molecular-weight fucoidan. All enrolled participants received conventional therapy for chronic pulmonary diseases and oral supplementation with OF at a dose of 550 mg per tablet, two tablets twice daily, for 12 weeks. No major protocol deviations, including changes in intervention dosage or study procedures, occurred during the study period. Participants were fully informed of the study purpose and procedures, and the study protocol was approved by the Institutional Review Board of Cheng Ching Hospital (HP2110034). All 46 participants completed the 12-week follow-up and were included in the final analyses. OF was administered as an adjunctive supplementation to standard medical treatment, and no placebo or untreated control group was included in this exploratory study. Adverse events were monitored throughout the study period through scheduled clinical visits and participant self-reporting. No serious adverse events related to oligo-fucoidan supplementation were observed. Any minor adverse events were transient and did not require discontinuation of the intervention. This clinical study was registered with the Thai Clinical Trials Registry (TCTR; registration number: TCTR20260126009; date of registration: January 26, 2026). The registration was completed after participant enrollment had begun, as this investigator-initiated exploratory study was not initially planned as a formal clinical trial requiring prospective registration.

### Assessment of PM_2.5_ exposure

Ambient PM_2.5_ exposure was estimated using data obtained from fixed-site air quality monitoring stations located near each participant’s residence. Baseline PM_2.5_ exposure was defined as the average PM_2.5_ concentration measured by the nearest monitoring station during the three days preceding the baseline blood sampling. This value was used to represent ambient exposure at study entry. During the intervention period, time-averaged PM_2.5_ concentrations were calculated for each follow-up interval to reflect cumulative ambient exposure. PM_2.5_ exposure at week 4 was defined as the average concentration measured from the initiation of the study to week 4. Exposure at week 8 was calculated as the average PM_2.5_ concentration measured between weeks 4 and 8, and exposure at week 12 was calculated as the average concentration measured between weeks 8 and 12. This approach was adopted to approximate participants’ ambient air pollution exposure over time and to align exposure windows with longitudinal biological measurements. Although personal exposure monitoring and detailed time–activity data were not available, the use of residential-area monitoring data represents a commonly applied method in environmental and clinical epidemiological studies to estimate ambient PM_2.5_ exposure.

### Analysis of basic and biochemical parameters of the subjects

Anthropometric measurements, including body weight, height, and blood pressure, were obtained from all participants using standardized, IRB-approved procedures. Venous blood samples were collected under fasting conditions into heparinized tubes for the assessment of biochemical and hematological parameters. Measured parameters included glucose, aspartate aminotransferase (AST), alanine aminotransferase (ALT), total cholesterol, triglycerides, albumin, white blood cell (WBC) count, red blood cell (RBC) count, platelet count, hemoglobin, creatinine, ferritin, and total bilirubin. Following collection, blood samples were processed to separate serum and cellular components. Serum aliquots were stored at − 80 °C until further analysis.

### Analysis of lymphocytes

Lymphocyte subsets were analyzed using flow cytometry with fluorochrome-conjugated monoclonal antibodies. Whole blood samples were aliquoted and incubated with specific antibodies for surface marker staining. Briefly, one aliquot of whole blood was incubated with fluorescein isothiocyanate (FITC)-conjugated anti-human CD19 antibody to identify B lymphocytes. A separate aliquot was incubated with phycoerythrin (PE)-conjugated anti-human CD3 antibody to identify total T lymphocytes. For T-cell subset analysis, additional aliquots of whole blood were stained with PE-conjugated anti-human CD3 antibody in combination with FITC-conjugated anti-human CD4 or FITC-conjugated anti-human CD8 antibodies. All staining procedures were performed for 20 min at 4 °C in the dark. A PE-conjugated mouse IgG1 antibody was used as an isotype control. All antibodies were purchased from BD Biosciences (Franklin Lakes, NJ, USA).

### Analysis of inflammatory cytokines

Frozen serum samples stored at − 80 °C were thawed prior to analysis. Serum concentrations of interleukin-6 (IL-6), tumor necrosis factor-alpha (TNF-α), and interleukin-8 (IL-8) were quantified using commercially available enzyme-linked immunosorbent assay (ELISA) kits according to the manufacturers’ instructions.

### Analysis of heavy metals

Serum samples were collected in contamination-free, acid-washed plastic tubes. Prior to analysis, samples underwent acid digestion. Briefly, 1 mL of serum was transferred into a digestion vessel, followed by the addition of 10 mL of ultrapure nitric acid. The mixture was heated at 60 °C on a hot plate for 30 min and subsequently heated to 95 °C until complete digestion was achieved and the solution became clear. After cooling to room temperature, the digested solution was transferred to a 25 mL volumetric flask. The digestion vessel was rinsed with deionized water, and the rinsing solution was added to the volumetric flask, which was then brought to volume with deionized water. The final solution was filtered through a membrane filter into a storage container prior to analysis. Blank samples were prepared using the same digestion procedure without serum to serve as procedural controls. Concentrations of heavy metals in the test samples, blanks, and standard solutions were determined using flame atomic absorption spectrometry (FAAS). Metal concentrations were calculated based on absorbance values and expressed as parts per million (ppm) according to standard calibration procedures.

### Statistical analysis

All results are presented as mean ± standard deviation (SD). Consistent with the exploratory, single-arm design of the study, statistical analyses were conducted primarily using descriptive methods. Temporal changes within each participant were summarized across baseline and weeks 4, 8, and 12, and subgroup trends according to ambient PM₂.₅ exposure were described narratively. No formal inferential hypothesis testing (e.g., t-tests, ANOVA, or p-values) was performed, as the study was not designed to establish causal effects or statistically confirmed longitudinal changes. All observed trends should be interpreted cautiously and considered as exploratory observations.

## Results

### Patient characteristics

A total of 46 participants were enrolled and received conventional treatment with adjunctive oligo-fucoidan (OF) supplementation (550 mg per tablet, two tablets twice daily for 12 weeks) **(**Fig. 1**)**. The distribution of chronic respiratory diseases among participants was as follows: chronic obstructive pulmonary disease (COPD; *n* = 20), asthma (*n* = 7), pulmonary fibrosis (*n* = 13), combined COPD and pulmonary fibrosis (*n* = 4), pulmonary embolism (*n* = 1), and bronchiectasis (*n* = 1). Baseline demographic characteristics and biochemical parameters of the participants, as well as their values over the 12-week study period, are summarized in Table 1. Descriptive changes in several biochemical parameters were observed over time. White blood cell count, glucose, alanine aminotransferase, and C-reactive protein (CRP) levels showed numerical differences at later time points compared with baseline; however, no statistically significant changes were detected across the study period. At baseline, CRP concentrations were within the moderate inflammatory range (1–10 mg/dL). During the study period, CRP concentrations were observed to be within the normal range (< 1 mg/dL) at week 12 compared with baseline values; however, this change did not reach statistical significance (*p* = 0.136).

**Table 1:** The characteristics of subjects before and during the supplementation period.

	baseline	4^th^week	8^th^week	12^th^week
Sex (male/female)	30/16			
**Age (y/o)**	64.89±10.3			
**BMI (kg/m** ^**2**^ **)**	24.26±6.19			
RBC (*10 ^**3**^**/μL)**	4.91±0.59	4.33±1.04	4.97±0.57	4.78±0.42
**Hb (g/dL)**	13.68±2.23	13.43±2.65	13.68±2.22	13.64±2.14
WBC(*10 ^**3**^ **/μL)**	6.98±1.87	6.6±1.83	6.8±2.17	6.57±2.04
Platelet(*10 ^**3**^ **/μL)**	233.58±66.89	223.28±61.55	228.78±53.73	230.26±59.74
**FPG (mg/dl)**	112.42±37.47	106.4±28.45	102.81±21.13	104.75±22.16
**AST (U/L)**	23.77±9.95	23.72±7.56	22.37±8.1	22.59±7.49
**ALT (U/L)**	22.04±15.72	19.95±9.87	20.34±9.8	19.11±8.32
**Cholesterol (mg/dL)**	177.93±38.67	180.5±38.43	171.72±33.85	173.19±35.44
**TG (mg/dL)**	134.71±71.89	144.67±89.74	123.41±54.38	130.7±53.83
**Albumin (g/dL)**	4.25±0.4	4.3±0.42	4.33±0.4	4.37±0.31
**Creatinine (mg/dL)**	1.09±1.07	0.94±0.87	0.93±0.84	1.04±0.97
**Ferritin(ng/dL)**	297.02±317.51	305.32±317.77	330.74±320.54	354.49±426.91
**T-Bilirubin (mg/dL)**	0.51±0.2	0.52±0.25	0.54±0.25	0.6±0.28
**CRP(mg/dL)**	0.65±1.56	0.41±0.62	0.38±0.57	0.23±0.29
**Lead(pb)(μg/dL)**	1.57±0.57	-	-	1.81±0.78
**Cadmium(cd)(μg/dL)**	1.3±0.19	-	-	1.29±0.23
**Mercury(Hg)(μg/dL)**	4.36±4.15	-	-	4.48±4.93

1. Values are expressed as mean ± SD.

2. FPG = Fasting Plasma Glucose; Hb= Hemoglobin; ALT = aspartate aminotransferase; ALT = alanine aminotransferase; TG = Triglyceride; CRP=C-reactive protein.

3. All data are presented descriptively; no inferential statistical testing (e.g., ANOVA) was performed.

### PM_2.5_ levels before and during the supplementation period

Average ambient PM_2.5_ concentrations during the study period are summarized in Table 2. At baseline, the mean PM_2.5_ concentration was 14.7 ± 10.3 µg/m³. Based on residential-area monitoring data, 15 participants were classified as having lower ambient PM2.5 exposure (0–11 µg/m³; “Good” group), with a mean concentration of 6.3 ± 2.8 µg/m³, whereas 31 participants were classified as having higher ambient PM_2.5_ exposure (> 12 µg/m³; “Slightly worse” group), with a mean concentration of 21.2 ± 9.2 µg/m³. Baseline PM_2.5_ values were numerically higher than those recorded at weeks 4, 8, and 12., particularly in the higher-exposure group. These observations are consistent with publicly available national air quality data reported by the Ministry of Environment’s Air Quality Monitoring Network in Taiwan, which indicate a general long-term downward trend in average PM2.5 levels in Taichung over recent years.

**Table 2:** The levels of PM_2.5_ before and during the supplementation period.

The stage of PM_2.5_	Baseline(μg/m^3^) (*n*=46)	4^th^week(μg/m^3^) (*n*=46)	8^th^week(μg/m^3^) (*n*=46)	12^th^week(μg/m^3^) (*n*=46)
Good	6.3±2.8(30)	8.8±1.9(30)	6.4±2.4(28)	6.8±2.6(29)
Slightly worse	21.2±9.2(22)	16.9±3.1(16)	16.6±3.5(18)	18.5±2.2(17)
Total stage	14.7±10.3	11.1±4.0	11.2±5.0	12.3±4.1

1. Values are expressed as mean ± SD.

2. The stages of PM_2.5_ : Good -0-12 μg/m^3^; Slightly worse-≥ 12 μg/m^3^.

### Changes in Lymphocyte Subsets During the Supplementation Period

The distributions of lymphocyte subsets during the supplementation period are presented in Table 3. Descriptive changes in lymphocyte subset proportions were observed over time. At week 12, the proportions of CD3⁺, CD3⁺CD4⁺, and CD3⁺CD8⁺ cells were numerically higher compared with baseline, whereas the proportion of CD19⁺ cells was numerically lower. These variations did not demonstrate statistically significant longitudinal changes and are therefore reported descriptively. The mean proportion of CD3⁺ cells increased by approximately 3.6%, CD3⁺CD4⁺ cells increased by approximately 2.05%, and CD19⁺ cells decreased by approximately 1.5% over the 12-week period; however, none of these changes reached statistical significance **(**Table 4**)**.

**Table 3:** The proportions of lymphocytes subtypes during the supplementation period.

	Baseline	4^th^week	8^th^week	12^th^week
CD3^+^	52.6±12.15	51.8±11.8	54.4±11.6	57.0±11.1
CD3^+^CD4^+^	32.5±9.0	31.5±8.7	33.0±9.3	34.7±7.7
CD3^+^CD8^+^	20.72±9.9	20.5±7.7	20.8±9.1	22.1±8.8
CD19^+^	11.9± 8.0	11.0± 6.9	11.7± 8.2	11.0± 7.5

1. Values are expressed as ± SD.

2. All data are presented descriptively; no inferential statistical testing (e.g., ANOVA) was performed.

**Table 4:** Changes in Lymphocyte Subsets During the Supplementation Period.

	4^th^week	8^th^week	12^th^week
△CD3^+^	-1.36±11.137	1.509±9.651	3.618±11.769
△CD3^+^CD4^+^	-1.029±6.919	0.603±7.639	2.053±8.598
△CD3^+^CD8^+^	-0.563±5.534	0.014±5.996	0.703±5.533
△CD19^+^	-0.67±3.737	-0.3±5.026	-1.535±5.098

1. Values are expressed as ± SD.

2. △: other therapy period groups- baseline group, for example: △CD3^+^CD4^+^ 4^th^week = CD3^+^CD4^+^ 4^th^week - CD3^+^CD4^+^ baseline.

3. All data are presented descriptively; no inferential statistical testing (e.g., ANOVA) was performed.

### Lymphocyte subset distributions stratified by ambient PM2.5 levels

Changes in lymphocyte subset proportions after 12 weeks, stratified by ambient PM_2.5_ exposure categories, are summarized in Table 5. Participants were categorized into lower-exposure (“Good”) and higher-exposure (“Slightly worse”) groups based on residential-area PM_2.5_ levels. In the lower-exposure group, numerical increases in the proportions of CD3⁺ and CD3⁺CD4⁺ cells were observed, whereas changes in CD3⁺CD8⁺ and CD19⁺ cells showed variable patterns. In the higher-exposure group, the proportions of CD3⁺, CD3⁺CD4⁺, and CD3⁺CD8⁺ cells were numerically higher at week 12 compared with baseline, while the proportion of CD19⁺ cells was numerically lower.

The mean proportion of CD3⁺CD4⁺ cells increased by approximately 3%, and the proportion of CD19⁺ cells decreased by approximately 3.13% in the higher-exposure group; however, these differences did not reach statistical significance. All subgroup comparisons are therefore reported descriptively.

**Table 5:** The changes of lymphocytes subtypes after 12-week supplementation in different PM_2.5_ stages.

	Good	Slightly worse
△CD3^+^	1.69±12.44	2.36±11.58
△CD3^+^CD4^+^	1.91±8.72	2.99±8.54
△CD3^+^CD8^+^	-0.14±10.51	1.64±8.78
△CD19^+^	1.56±6.61	-3.13±8.14

1. Values are expressed as ± SD.

2. △: other therapy period groups- baseline group, for example: △CD3^+^CD4^+^ 4^th^week = CD3^+^CD4^+^ 4^th^week - CD3^+^CD4^+^ baseline.

3. Data are presented descriptively; no inferential statistical tests were performed.

4. The stages of PM_2.5_ : Good -0-12 μg/m^3^; Slightly worse-≥ 12 μg/m^3^.

### Serum heavy metal levels stratified by ambient PM_2.5_ exposure

Serum heavy metal concentrations were assessed descriptively during the study period, and results stratified by ambient PM_2.5_ exposure categories are presented in Table 6. At baseline, serum cadmium (Cd) and mercury (Hg) concentrations were numerically higher in the higher-exposure (“Slightly worse”) group than in the lower-exposure (“Good”) group. After 12 weeks, Cd and Hg concentrations in the higher-exposure group appeared numerically lower compared with baseline; however, these changes did not reach statistical significance. Accordingly, differences in serum heavy metal levels between time points and exposure groups are reported descriptively.

**Table 6:** The levels of heavy metals after supplementation in different PM_2.5_ stages.

	Good		Slightly worse	
	Baseline	12^th^week	Baseline	12^th^week
Lead (pb)ug/dl	1.18± 1.2	1.2±1.79	0.7± 0.7	1.03±1.1
Cadmium(cd)ugl/l	0.12± 0.36	0	0.16± 0.51	0.1±0.4
Mercury(Hg)	1.18± 1.78	2.53±1.42	4.92± 4.33	3.05±2.27

1. Values are expressed as ± SD.

2. Data are presented descriptively; no inferential statistical tests were performed.

3. The stages of PM_2.5_ : Good -0-12 μg/m^3^; Slightly worse-≥ 12 μg/m^3^.

### Changes in inflammatory cytokine levels during the study period

**Fig. 2:**
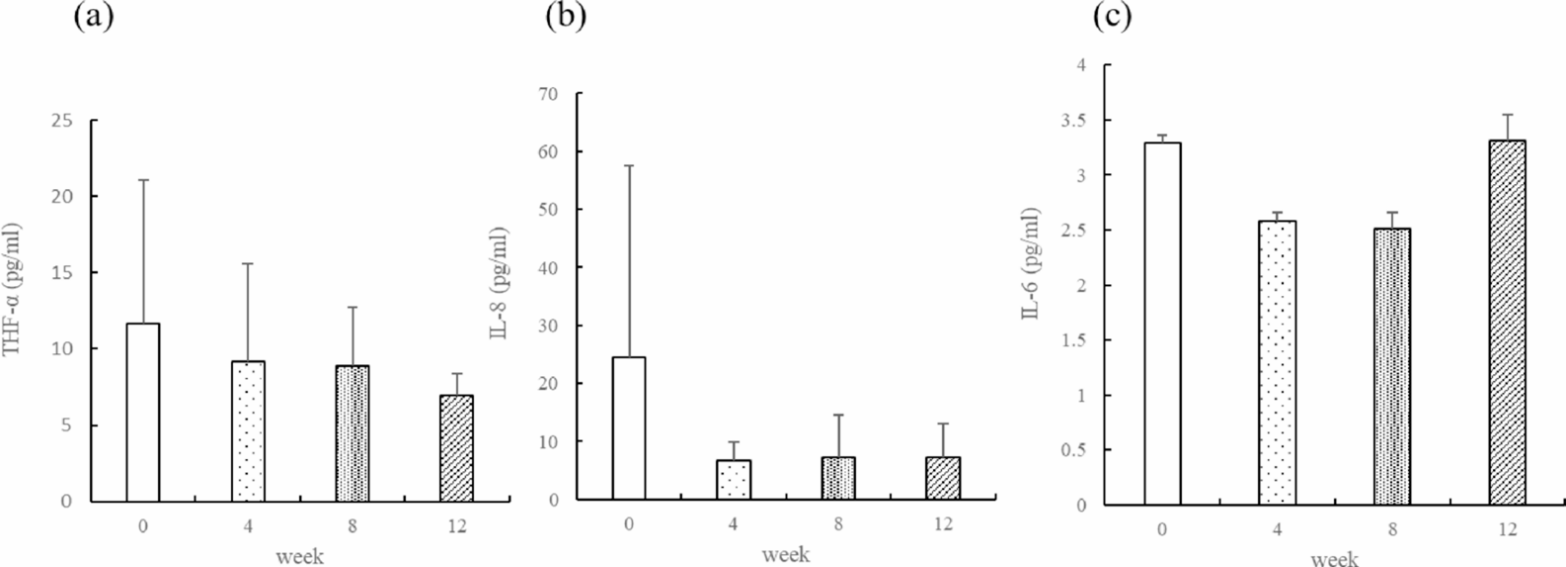
The concentrations of cytokines (**a**) TNF-α, (**b**) IL-8, (**c**) IL-6 over 12 weeks of oligo-fucoidan supplementation. Data are presented as mean ± SD at baseline and weeks 4, 8, and 12. Error bars represent standard deviation. All values are descriptive and should be interpreted with caution given the single-arm, exploratory design of the study. No inferential statistical testing was performed.

**Fig. 3:**
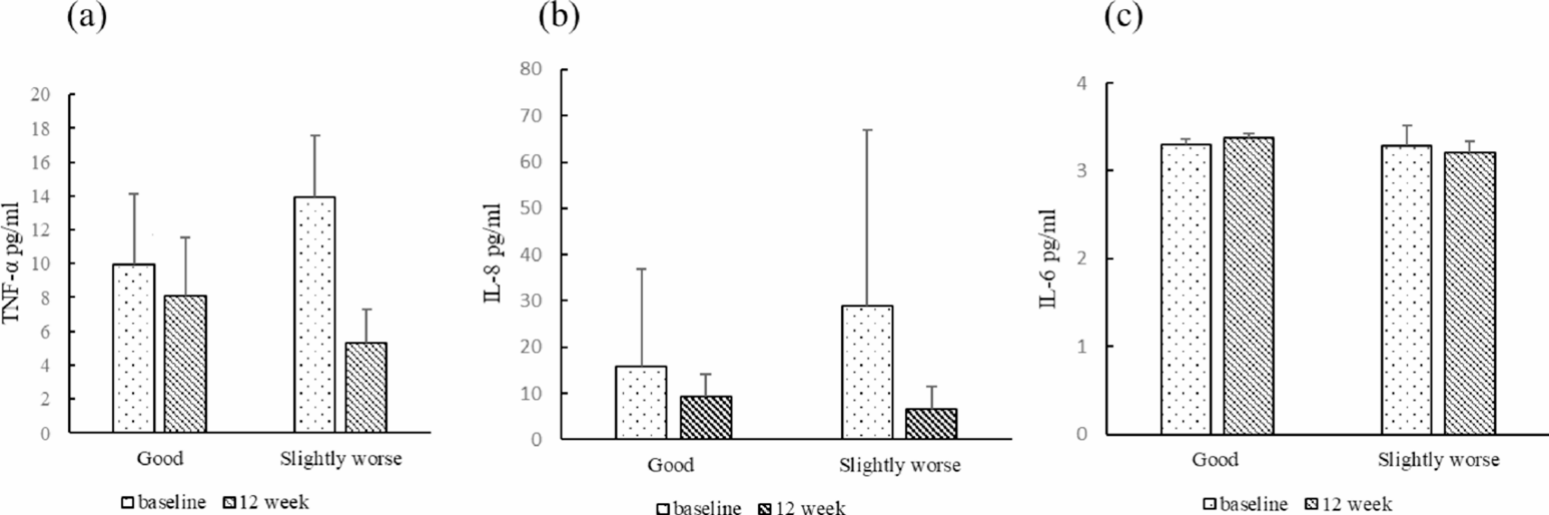
The changed concentrations of cytokines (**a**) TNF-α (**b**) IL-8 (**c**) ) IL-6 according to ambient PM₂.₅ exposure levels (lower-exposure “Good” vs. higher-exposure “Slightly worse” groups) at baseline and week 12. Values are presented as mean ± SD. Error bars represent standard deviation. Observed differences between groups and over time are descriptive only and should be interpreted with caution. No inferential statistical testing was performed.

Serum concentrations of inflammatory cytokines, including tumor necrosis factor-alpha (TNF-α), interleukin-8 (IL-8), and interleukin-6 (IL-6), were measured at baseline and at weeks 4, 8, and 12 (Fig. 2). Over the 12-week study period, IL-8 values at later time points were lower than baseline, TNF-α values at later time points were lower than baseline, and IL-6 values remained relatively stable. When participants were stratified according to ambient PM₂.₅ exposure levels (lower-exposure “Good” group vs. higher-exposure “Slightly worse” group), both TNF-α and IL-8 values at week 12 were generally lower than at baseline in both exposure groups (Fig. 3). In the higher-exposure group, TNF-α values at week 12 were lower than in the lower-exposure group. IL-8 values in the lower-exposure group were lower at later time points compared with baseline. These observations are presented descriptively and should be interpreted with caution given the exploratory, single-arm nature of the study.

### Discussion

This exploratory, single-arm clinical study examined immunological, inflammatory, and metal-related biological profiles in patients with chronic respiratory diseases residing in environments affected by ambient PM₂.₅ pollution and receiving adjunctive oligo-fucoidan supplementation. Rather than establishing causal effects, the study aimed to descriptively characterize longitudinal changes in immune cell distributions, inflammatory cytokines, and serum heavy metal concentrations within a real-world clinical context of ongoing standard therapy. During the study period, ambient PM₂.₅ exposure levels among participants were predominantly within the “good” to “fair” range according to Taiwan’s air quality standards, with levels generally remaining within a relatively low range during the study period. These exposure patterns were consistent with contemporaneous regional air quality data reported by Taiwan’s environmental monitoring network, reflecting a gradual decline in average PM₂.₅ concentrations in the Taichung area. Accordingly, PM₂.₅ exposure in this study should be interpreted as a background environmental context rather than as a high-intensity exposure scenario. Stratification into lower- and higher-exposure groups was therefore intended to provide descriptive comparisons rather than to support exposure–response inference.

PM₂.₅ is a complex mixture containing redox-active components, including transition metals and organic compounds, which have been implicated in oxidative stress, immune dysregulation, and chronic inflammatory processes. Chronic exposure has been associated with altered cytokine profiles and shifts in lymphocyte subsets, reflecting sustained immune activation rather than acute toxicity. In this context, the biomarkers selected in the present study—including inflammatory cytokines, T- and B-cell populations, and serum heavy metal concentrations—were chosen to capture downstream biological responses relevant to environmental toxicology and chronic respiratory disease, rather than to interrogate specific molecular pathways.

With respect to lymphocyte subsets, descriptive changes were observed over the 12-week period, including numerically higher proportions of T-cell populations and lower proportions of CD19⁺ B cells, particularly among participants residing in higher ambient PM₂.₅ environments. Although these changes did not reach statistical significance, similar patterns have been described in prior studies of chronic air pollution exposure. Prior studies have suggested that oxidative stress–driven pulmonary injury may influence adaptive immune balance, including B-cell–related inflammatory responses. In the present study, hyperoxia-related literature was referenced not as a direct exposure analogue to PM₂.₅, but as a conceptual model illustrating shared downstream mechanisms of oxidative stress–mediated immune dysregulation in lung tissue. Hyperoxia and PM₂.₅ represent distinct environmental insults^33^; however, both have been shown to induce oxidative stress, inflammatory signaling, and immune cell redistribution in pulmonary systems. The citation of hyperoxia models was therefore intended to contextualize potential biological plausibility rather than to equate exposure mechanisms. Importantly, the immune cell changes observed in this study should be interpreted as exploratory associations rather than evidence of a specific immunomodulatory effect.

Serum heavy metal concentrations exhibited substantial interindividual variability and did not demonstrate statistically significant longitudinal changes at the group level. Given the long biological half-lives of metals such as cadmium and mercury, the 12-week follow-up period may have been insufficient to detect meaningful changes in systemic concentrations. While prior experimental studies have suggested that fucoidan have been suggested in experimental settings to possess potential metal-binding or antioxidative properties, the present findings do not provide definitive evidence of metal reduction in humans. Rather, they offer descriptive data on metal-related biological profiles in patients with chronic respiratory diseases residing in air pollution–affected environments. Previous experimental studies have reported that fucoidan may interact with certain metals through chelation or antioxidative mechanisms^34–36^; however, whether such effects translate into measurable reductions in systemic metal burden in clinical populations remains uncertain and warrants further investigation.

Regarding inflammatory cytokines, IL-8 values at later time points were lower than baseline, TNF-α values showed descriptive reductions, and IL-6 values remained relatively stable. IL-8 is a key neutrophil chemoattractant implicated in chronic airway inflammation and has been reported to respond to environmental particulate exposure. Experimental studies have suggested that airborne particulate matter may stimulate lung epithelial cells through activation of NF-κB and mitogen-activated protein kinase (MAPK) pathways, including p38, ERK, and JNK, thereby increasing TNF-α and IL-8 expression^17,37–39^. In the present study, the descriptive changes in IL-8 are presented as temporal observations, the biological relevance of which remains uncertain in the absence of controlled comparison.; however, in the absence of a control group and given the influence of concomitant medications, these observations should be interpreted with caution. Subgroup analyses stratified by ambient PM₂.₅ exposure were descriptive in nature and were not intended to indicate differential treatment effects. Although molecular signaling pathways were not directly assessed, prior experimental evidence suggests that fucoidan may influence inflammatory processes, potentially through modulation of NF-κB–related pathways. Whether similar mechanisms operate in patients with chronic respiratory diseases remains to be determined. Accordingly, the current findings should be regarded as exploratory observations rather than direct mechanistic evidence in this population.

Several important limitations warrant consideration. First, the non-randomized, open-label, single-arm design without a parallel control group limits causal inference and increases susceptibility to selection bias and residual confounding. Second, the modest sample size and inclusion of heterogeneous chronic respiratory diseases, including COPD, asthma, and pulmonary fibrosis, likely contributed to biological variability and precluded disease-specific analyses. Third, the 12-week duration may have been insufficient to capture long-term changes in chronic inflammatory markers and heavy metals with prolonged biological persistence. In addition, air pollution exposure was estimated using residential-area ambient monitoring data, which may not accurately reflect individual-level exposure influenced by mobility, occupational settings, indoor air quality, and time–activity patterns. Averaging PM₂.₅ concentrations over broad time windows may have further attenuated exposure contrasts and biological associations. Furthermore, participants continued standard medical therapies, including corticosteroids and bronchodilators, which are known to affect immune and inflammatory markers. Although treatment regimens were clinically stable, the absence of medication adjustment limits attribution of observed changes to supplementation alone. Finally, no a priori power calculation was performed, as the study was designed as a feasibility-driven exploratory investigation; consequently, the study may have been underpowered to detect modest effects.

Despite these limitations, this study provides preliminary human data linking ambient air pollution exposure, immune-related biomarkers, and inflammatory profiles in patients with chronic respiratory diseases under real-world clinical conditions. The findings should be viewed as hypothesis-generating and may inform the design of future randomized controlled trials incorporating personal exposure assessment, longer follow-up durations, medication-adjusted analyses, and clinically meaningful outcomes such as pulmonary function and symptom burden.

## Conclusions

This exploratory study provides preliminary clinical evidence that adjunctive oligo-fucoidan supplementation is associated with descriptive changes in immune and inflammatory profiles in patients with chronic respiratory diseases residing in air pollution–affected environments. While the findings should be interpreted cautiously due to the non-randomized design and limited sample size, they offer valuable human data relevant to the interface between environmental exposure, immune regulation, and chronic respiratory disease. Importantly, this study does not establish causal relationships or definitive longitudinal effects. Rather, it characterizes immune- and inflammation-related biomarkers under real-world clinical conditions and provides a foundation for future large-scale, controlled clinical trials and mechanistic investigations aimed at clarifying the clinical relevance and potential role of oligo-fucoidan as an adjunctive intervention.

## Data Availability

The data that support the findings of this study are available from the corresponding author, upon reasonable request.
